# Retraction Note: Down-regulation of miR-133a and miR-539 are associated with unfavorable prognosis in patients suffering from osteosarcoma

**DOI:** 10.1186/s12935-016-0359-5

**Published:** 2016-11-04

**Authors:** Alireza Mirghasemi, Afshin Taheriazam, Seyyed Hasan Karbasy, Ali Torkaman, Mohammadreza Shakeri, Emad Yahaghi, Aram Mokarizadeh

**Affiliations:** 1 Department of Orthopedics, Qom University of Medical Sciences, Qom, Iran; 2 Department of Orthopedics Surgery, Tehran Medical Sciences Branch, Islamic Azad University, Tehran, Iran; 3 Department of Anesthesiology, Birjand University of Medical Sciences, Birjand, Iran; 4 Department of Orthopedics, Firoozgar Hospital, Iran University of Medical Sciences, Tehran, Iran; 5 Department of Orthopaedic and Trauma Surgery, Birjand University of Medical Sciences, Birjand, Iran; 6 Department of Molecular Biology, Baqiyatallah University of Medical Sciences, Tehran, Iran; 7 Cellular & Molecular Research Center, Kurdistan University of Medical Sciences, Sanandaj, Iran

## Retraction to: Cancer Cell Int (2015) 15:86 DOI 10.1186/s12935-015-0237-6

The Editors-in-Chief and Publisher have retracted this article [1] because the scientific integrity of the content cannot be guaranteed. An investigation by the Publisher found it to be one of a group of articles we have identified as showing evidence suggestive of attempts to subvert the peer review and publication system to inappropriately obtain or allocate authorship. This article showed evidence of plagiarism (most notably from the articles cited [2, 3]) and authorship manipulation.
